# Human Genetic Markers and Structural Prediction of* Plasmodium falciparum* Multidrug Resistance Gene* (pfmdr1)* for Ligand Binding in Pregnant Women Attending General Hospital Minna

**DOI:** 10.1155/2018/3984316

**Published:** 2018-05-16

**Authors:** B. Lawal, O. K. Shittu, A. Abubakar, A. Y. Kabiru

**Affiliations:** Department of Biochemistry, Federal University of Technology, P.M.B. 65, Minna, Nigeria

## Abstract

The study aims to determine the association of malaria infection with ABO blood groups and genotype and also to detect point mutations at positions 86, 184, 1034, and 1042 of the* Plasmodium falciparum* multidrug resistance gene* (pfmdr1)* in blood samples collected from pregnant women attending General Hospital Minna. Out of 250 pregnant women screened, 39 (15.60%) had malaria infection. Prevalence was higher in women, during the third trimester (46.15%), genotype AA (64.10%), and O blood group (53.84%) individuals when compared with others. There was significant (*p* < 0.05) decrease in Packed Cell Volume (PCV), hemoglobin (HGB), Red Blood Cells (RBC), and platelet (PLC) count in infected group when compared with noninfected group. Although, two of the isolates showed disrupted protein sequence at codon 1034–1042, no mutation was found in any of the* P. falciparum* isolates. Structural prediction of chemical ligand led to the identification of Neu5Ac*α*2-3Gal*β*1-3/*β*1-4Glc/GlcNAc. This compound can theoretically bind and change the functional integrity of the pfmdr1 protein, thus providing a new window for malaria drug target.

## 1. Introduction

Malaria is a vector borne infectious disease endemic in Nigeria with about 97% of the population at risk [1]. In Nigeria, malaria accounts for 60% of outpatient visits to health facilities, 25% of childhood death, and 11% of maternal death [2]. The financial loss due to malaria annually is estimated to be about 132 billion naira in form of treatment cost, prevention, and loss of man hours, among others [3]. Increased resistance of the* Plasmodium falciparum *parasite to many of the available antimalarial drugs has made the treatment of malaria increasingly difficult and poses a major threat to the malaria control and eradication program running across the globe [4]. The factors influencing the rate of spread of antimalarial drug resistance include de novo mutation, human population movement and infection among migrants, drug use, and malaria transmission intensity [5]. More than any other issue, poverty, in adherence to drug prescription, and inadequate access to drugs continue to be major force in the development of resistance. A wide range of unrecommended antimalarial medicines are available and sold over the counter in medicine stores and are accessible to multigravid pregnant women in Nigeria [1]. Healthcare providers also prescribed unrecommended antimalarials due to poor knowledge on best practices and low confidence in the recommended drugs. The impact of antimalarial drug resistance includes high mortality (resistant infections are more often fatal) and morbidity. Resistance to antimalarial drugs has also increased the global cost of controlling the disease and investigations on newer and more expensive drugs. Therapeutic failure necessitates consultation at a health facility for further diagnosis and treatment, resulting in loss of working days for adults and absence from school for children.

Malaria during pregnancy is a major public health concern. In most endemic areas, pregnant women are the main adult risk group for malaria [6]. Pregnant women are particularly susceptible to severe malaria and have a greater risk of antimalaria resistance due to loss of immunity during pregnancy, thus placing them at high risk of maternal and infant morbidity and mortality, abortion, premature labour, and stillbirth [7].

Genetic markers such as hemoglobin genotypes and blood groups have been associated with malaria but have been implicated with rosette formation and cytoadhesion [8]. Variations in gene encoding functional glycotransferase have been associated with protection from* P. falciparum *malaria. Also trisaccharide of “A” and “B” blood group is presumed to act as receptor and function as an important factor for rosetting. However, the RBC of group O does not express trisaccharide blood types [9].

Genetic variations in* pfmdr1* gene have played vital role in malaria chemotherapy. Mutations (N86Y, Y184F, S1034C, N1042D, and D1246Y) in the* pfmdr1* gene have been associated with resistance to multiple antimalarial drugs such as quinine, mefloquine, halofantrine, artemisinin, lumefantrine, CQ, and amodiaquine [10–13]. However, mutations in this gene have been associated with geographic areas [14], and there is a paucity of information on antimalarial drug resistance markers in* Plasmodium falciparum* infected pregnant women in Northern Nigeria. Therefore, it becomes relevant to determine the molecular marker of resistance in populace of this region. Also, understanding the relationship between* pfmdr1* amino acid sequence and its three-dimensional structures will assist in predicting the chemical ligands/inhibitor that can bind and change the functional integrity of the proteins. This will provide information that is crucial in designing an alternative and effective antimalarial drug, thus providing a new window for malaria drug target.

## 2. Materials and Methods

### 2.1. Study Area

This study was carried out in Minna, Niger State located in the tropical zone of North Central Nigeria. Minna, the capital of Niger State, Nigeria, is located within longitude 6°33′E and latitude 9°37′N, covering a land area of 88 km^2^ with a population of 1.2 million (2006 Population Census). Minna has a tropical climate with mean annual temperature, relative humidity, and rainfall of 30.20°, 61.00%, and 1334.00 cm, respectively. The climate presents two distinct seasons: a rainy season (April–October) and dry season (November–March).

### 2.2. Inclusion and Exclusion Criteria

All women enrolled had earlier received intermittent preventive treatments (IPTp) with sulphadoxine-pyrimethamine (SP), as per current practice. The eligibility factor was set as women presenting with gestational age 4 weeks and above to ensure adequate coverage and data control. Those who were not resident in the study area, visitors, and temporary residents who were not officially registered for antenatal were not eligible for enrolment. Other exclusion criteria included history of adverse drug reaction or severe disease (such as hepatitis, jaundice, tuberculosis, and obvious AIDS symptoms).

### 2.3. Ethical Considerations

This study was approved by the Ethical Committee of the Niger State Ministry of Health, Minna, Nigeria. Oral consent was obtained from the participant prior to inclusion in the study and before sample collection. All procedures were performed according to the guidelines for human experimentations in clinical research as stated by the Federal Ministry of Health of Nigeria.

### 2.4. Experimental Subjects and Sample Collection

A total of 250 women that attended antenatal care during the period of study (December 2016) were recruited. Each pregnant woman was finger pricked using a sterile lancet to obtain capillary blood for a rapid diagnostic test (RDT) for malaria. Safety procedures were adopted in the collection of finger-prick blood samples by swabbing the area to be sampled with 70% alcohol and allowing it to dry before collection. Two milliliters (2 mL) of blood was then drawn (venipuncture) from malaria positive sample with a sterile disposable.

### 2.5. Parasite Detection

RDT based* P. falciparum *specific histidine-rich protein-2 (Bioline, USA) was performed according to the manufacturer's instructions to detect parasites as recommended by the National Malaria Programme [15].

### 2.6. ABO Blood Grouping and Hemoglobin Genotyping

The ABO blood group of each subject was determined using cell grouping antisera (A, B, and D) as described by Simon-Oke et al., [16], while the hemoglobin genotype separation was carried out using cellulose acetate electrophoresis method as described by Cheesbrough, [17].

### 2.7. Hematological Parameters

The hematological parameters, hemoglobin (Hb), Packed Cell Volume (PCV), Red Blood Cells (RBC), White Blood Cells (WBC), and platelets count (PLT), were determined using the automated hematologic analyzer SYSMEX KX21, a product of SYSMEX Corporation, Japan, as described by Dacie and Lewis, [18].

### 2.8. Molecular Studies

#### 2.8.1. DNA Extraction

The genomic DNA was extracted from 2 ml of infected blood using QIAmp DNA Mini Kit (Qiagen, Hilden, Germany) according to the manufacturer's instructions and subsequently stored at −20°C until use. The presence of DNA was ascertained by subjecting it to 1% agarose gel electrophoresis (DNA GelRed™) and the purity of DNA was checked spectrophotometrically by calculating the* A*260/*A*280 ratio.

#### 2.8.2. Amplification of* pfmdr1* Gene

Amplification of the* pfmdr1* gene was performed on two fragments using polymerase chain reaction (PCR): the first fragment contained codons 86 and 184; the second fragment contained codons 1034 and 1042. The primers were designed on the basis of the* Plasmodium falciparum* 3D7 multidrug resistance protein (pfmdr1), partial mRNA sequence (GenBank accession no. XM_001351751.1). The reaction primers and conditions for amplification of the first fragment (580 bp) were Forward: AGGTTGAAAAAGAGTTGAAC and Reverse: ATGACACCACAAACATAAAT; reaction conditions were 94°C for 3 minutes/[94°C for 30 seconds, 45°C for 1 minute, 72°C for 1 minute] × 30 cycles/72°C for 5 minutes. The reaction primers and conditions for amplification of the second fragment (234 bp) were Forward: GCATTT TATAATATGCATACTG and Reverse: GGATTTCATAAAGTCATCAAC; reaction conditions were 94°C for 3 minutes/[94°C for 30 seconds, 55°C for 1 minute/65°C for 40 seconds] × 30 cycles/65°C for 5 minutes/15°C for 5 minutes [1, 13].

The PCR amplification reactions were performed in a 20-*μ*l volume PCR tube containing 1.0 U of Taq DNA polymerase (Pyro Hot Start), 250 *μ*M each of the dNTPs, 1x reaction buffer with 1.5 mM MgCl_2_, 1x stabilizer and tracking dye, and 4 *μ*l of genomic DNA. All PCR reactions were carried out in a thermal cycler (Techne TM Thermal cycler TC-312, Fisher Scientific, UK). PCR products were separated on a 1.5% agarose gel (Invitrogen) and stained with ethidium bromide for confirmation of amplification of the two* pfmdr1* fragments as indicated by a band at 580 bp and 234 bp.

#### 2.8.3. Sequencing Protocol for* pfmdr1*

Polymorphisms in the* pfmdr1 *gene were determined by direct sequencing of the amplicons resulting from the nested PCR using each primer for target gene amplification. The sequencing was done using BigDye Terminator v3.1 cycle sequencing kit in an ABI 3730 sequencer (Applied Biosystems). The deduced amino acid sequences were aligned and analyzed with the Lasergene^®^ software (DNASTAR, Madison, WI, USA) using the reference sequences of 3D7 retrieved from* Plasmodium* database (http://www.plasmodb.org).

### 2.9. Bioinformatics Studies

#### 2.9.1. Database Search

The similarities in sequenced PFMDR gene were compared using pairwise alignment tools (https://www.ncbi.nlm.nih.gov) and the nucleotides sequence was transcribed using sequence translation Transeq (EMBOSS) (https://www.ebi.ac.uk/tools/st). This was compared with databased sequences using basic local alignment search tools (BLASTP) search default setting (https://www.ncbi.nlm.nih.gov). Five hits were selected for multiple sequences alignment using Clustal Omega method BLOSUM matrix (http://www.eb.ac.uk/msa/tools/clustalo). Specific features such as protein family, domain, cellular component, biological process, and molecular function was predicted using InterPro (http://www.ebi.ac.uk/interpro).

#### 2.9.2. Receptor Protein Selection

The PDBSum (http://www.eb.ac.uk/pdbsum/) and* SAS* databases were searched using IPFMDR sequence to download the similar protein sequence for structural prediction. The best three hits were subjected to multiple sequence alignment (http://www.tcoffee.crg.cat) (Laskowski, 2001). The selected structure exists in chains. The chains A were retrieved from http://www.rcsb.org for the clustering and helical shapes alignment using ESPipt 3.0. Advanced and all the hits proteins were model PyMOL (http://www.pymol.org).

### 2.10. Statistical Analysis

Data generated from this study were analyzed using statistical package for social science (SPSS) version 16 and presented as means ± SE of the mean. Comparisons between different patient groups were carried out by *t*-test. The level of significance was set at *p* < 0.05.

## 3. Results

### 3.1. ABO Blood Groups and Genotype Distributions

Out of 250 pregnant women that were screened for malaria, 39 (15.60%) were found to be infected with* Plasmodium *parasites as determined by RDT. The prevalence of malaria was highest among third trimester (46.15%) as compared with the first (30.76%) and second trimester (23.07%) groups (Table 1). These differences were observed to be statistically significant (*p* = 0.039). All pregnant women that were positive for malaria were also tested for ABO blood (Table 2) groups and genotype (Table 3). The prevalence was higher among genotype AA (64.10%) than AS (35.89%) and this difference was not statistically significant (*p* = 0.206). The distribution of ABO phenotypes among malaria infection was observed not to be statistically significant (*p* = 0.09). The highest proportion was among individuals with O blood group (53.84%), followed by those with blood group B (25.64%), A (17.24%), and AB (7.69%)

### 3.2. Hematological Parameters

The levels of PCV, RBC, HGB, TWBC, and PLC in malaria infected and noninfected pregnant women attending General Hospital Minna are shown in Table 4. Test of significance (paired sample *t*-test) showed a significant differences in PCV and platelet count between malaria positive and malaria negative pregnant women (*p* < 0.05). The *p* value = 0.0243 and 0.007 for PCV and platelet count indicating a very significant decrease among malaria infected pregnant women.

### 3.3. Prevalence of Drug Resistant Molecular Markers

The genomic DNA of the 39 blood samples from malarial infected pregnant women were successfully isolated. However, out of the 39 genomic DNA samples, only 16 samples from infected women with resistance malaria were genotype for* pfmdr1* gene by PCR and nested PCR, out of which 12 (75%) were successful while 4 (25%) failed. Thus pregnant women with antimalarial resistance had* pfmdr1* gene prevalence of 75%. Amplification of the* pfmdr1* gene was performed in two different fragments. Seven (7) samples were successfully amplified in the first fragment (580 bp) containing codons 86 and 184; six (6) samples were amplified from second fragment (234 bp) containing codons 1034 and 1042.

### 3.4. *pfmdr1* Gene Sequence Data

No mutation was observed at codon positions 86, 184, 1034, and 1042 of* pfmdr1* in any of the* P. falciparum* isolates. However, two of the isolates from the second fragment (sequence for 1034 and 1042) showed disrupted protein sequence (Table 5)

### 3.5. Bioinformatics Database Search

**Figure 1:**
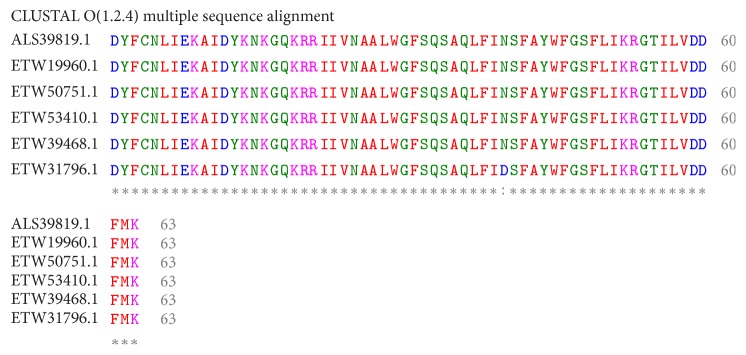
Similarities of the isolated* pfmdr1* gene with those reported from other African countries.

The amino acids and translated protein sequence of the of* pfmdr1* gene isolated from pregnant women attending General Hospital Minna are shown in Table 6. Although pairwise alignment of these two genes shows high (88%) percentage similarities and 3% gap between the sequences (Figure 5), BLASTP search of the second gene showed no significant similarity; however, the first gene produced multiple hits, out of which five (5) hits were selected for multiple sequence alignment. The results show very high similarities of the gene with those reported from other African countries (Figure 1).

### 3.6. Receptor Protein Selection

The PDBSum search of IPFMDR sequence retrieves seven (7) hits of similar protein sequence for structural prediction (Table 7). However, 4RHS, SPLTB, STT, and 5LUQ are the most similar protein with 31.7 and 34.8 percentages.

**Figure 2:**
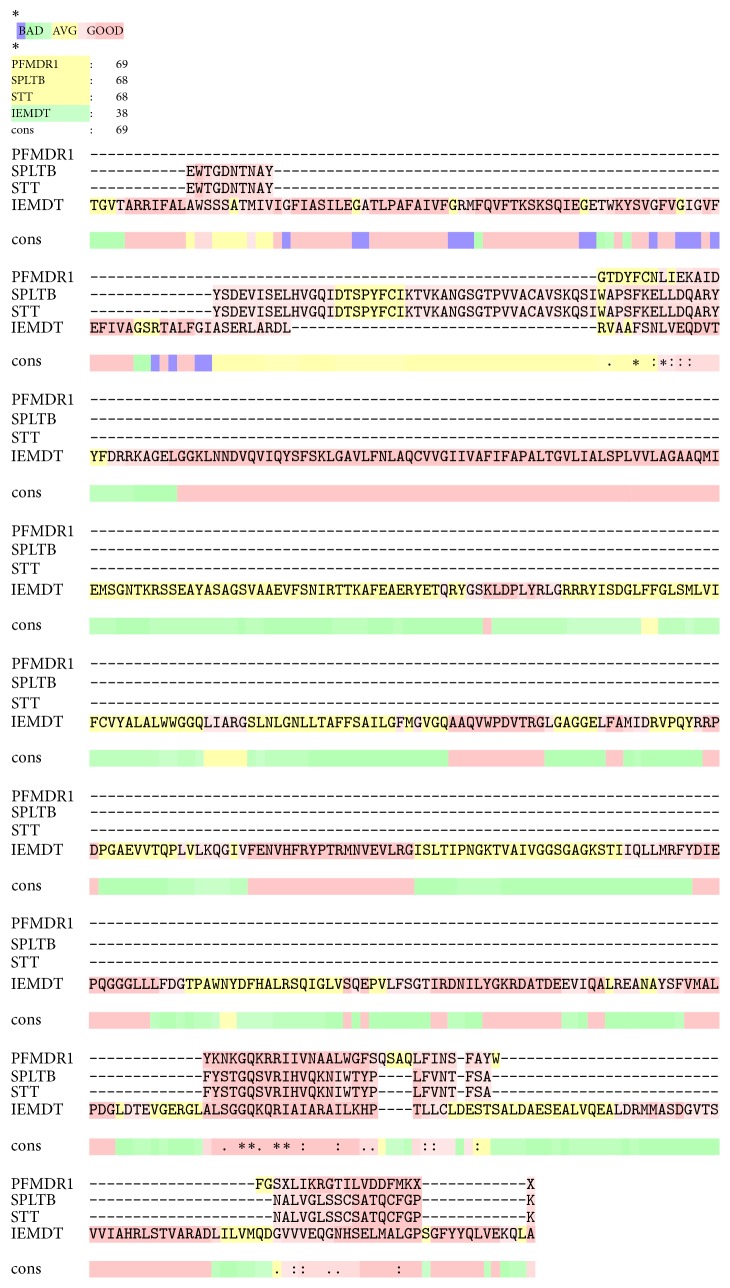
T-Coffee multiple sequences alignment of* pfmdr1* homologous protein.

**Figure 3:**
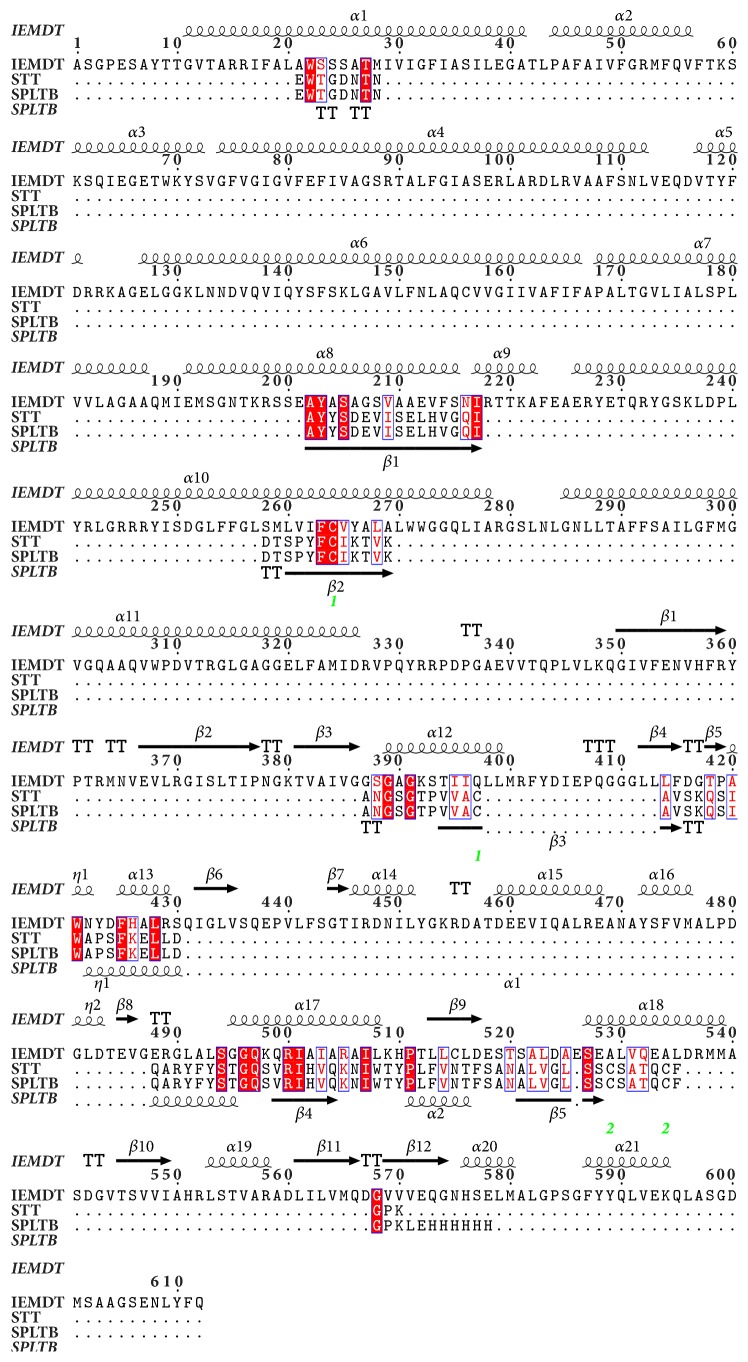
Clustering and helical shapes alignment of* pfmdr1* gene homologous protein. Included proteins are crystal structure of an inward-facing eukaryotic ABC multidrug transporter, crystal structure of pltb, and structure of typhoid toxin.* The secondary structure elements are as follows: α-helices are shown as large coils, 3*_*10*_* helices are shown in small coils labeled η, β strands are shown in arrows labeled β, and β turns are labeled TT. The identical residues are shown on a red background with conserved residues in red and conserved regions in blue boxes*.

**Figure 4:**
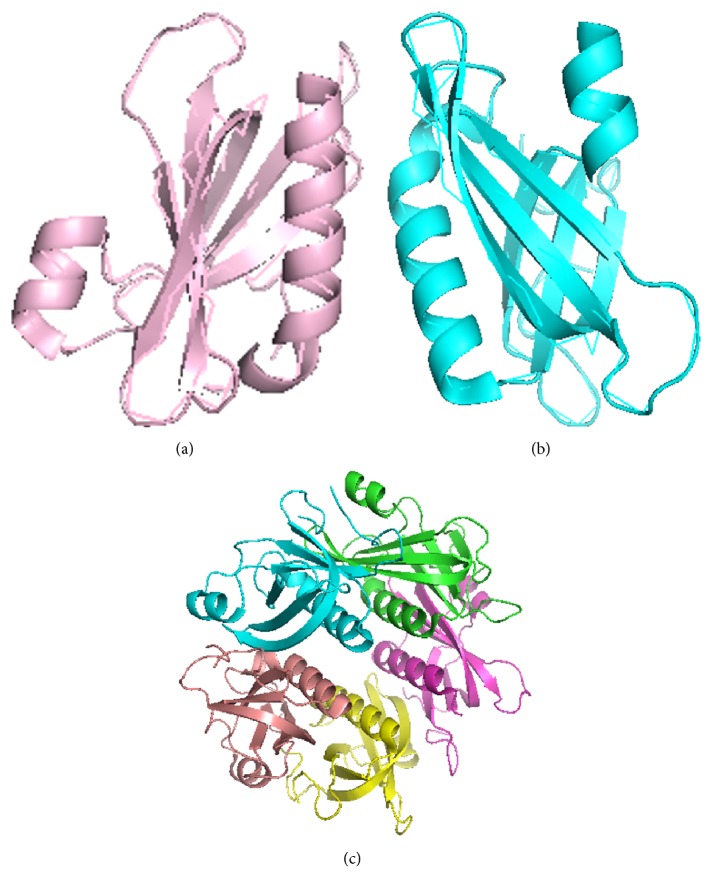
PyMOL build structure of (a) STT monomers, (b) SPLTTB monomer, and (c) SPLTTB pentamer.

**Figure 5:**
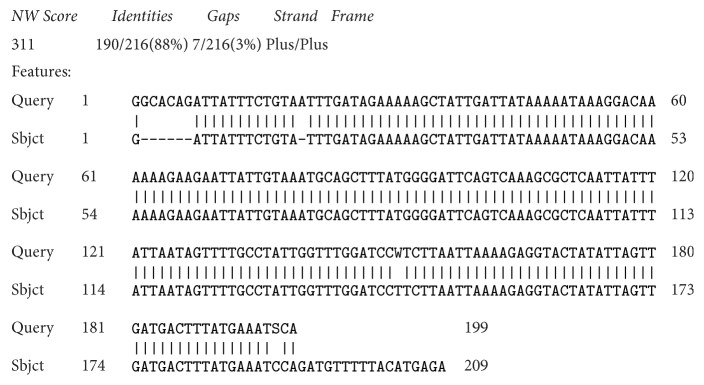
Pairwise alignment of the isolated* pfmdr1* gene sequences.

T-Coffee and ESPript 3.0 show multiple sequences alignment of* pfmdr1* homologous protein of crystal structure of an inward-facing eukaryotic ABC multidrug transporter (IEMDT), crystal structure of pltb (SPLTB), and structure of typhoid toxin (STT). The T-Coffee alignment shows percentage similarity of homologous protein IEMDT, SPLTB, and STT to be 37, 64, and 65, respectively (Figure 2), while ESPript 3.0 shows the 3-dimensional helical folding of the homologous protein (Figure 3).* IEMDT* has twenty-one *α*-helices, twelve *β* strands, and two n, while SPLTB and STT have two *α*-helices and five *β* strands (Figure 4).

## 4. Discussion

Pregnant women living in malaria endemic regions, particularly in sub-Saharan Africa are associated with a high frequency and density of* Plasmodium falciparum *parasite with high rates of maternal morbidity [19]. The prevalence of malaria demonstrated in this study was 15.60% lower than 52% and 88.2% reported among pregnant women attending antenatal clinic in southwest Nigeria [20, 21].

Previously, the reports of the prevalence of malaria in pregnancy were variable and high in Niger State where prevalence rates between 58.2% and 83.40% were documented [22, 23]. High prevalence of malaria in pregnancy (41%) has been attributed to poor compliance to the use of insecticide treated nets and intermittent preventive therapy [24]. However, these reports contrast sharply with the findings of this study where a prevalence rate of 15.60% was obtained for the pregnant women attending General Hospital Minna. This finding is not surprising as all women enrolled in this study had earlier received IPTp with SP, as per current practice in Niger State. Previous researchers have also shown strong correlation between the use of long lasting insecticide treated nets (LLIN) and reduction in prevalence of parasitaemia and anaemia in pregnant women, stillbirths, and improvements in birth weights of babies [25, 26]. Thus the low prevalence rate recorded in this study may be attributed to the improved understanding and compliances of the antenatal clinic women about malaria control strategies like use of LLIN and/or alternative intermittent preventive treatment with pyrimethamine sulfadoxine (SP). In addition, the lower prevalence obtained in this study might be due to the fact that this study was carried out during the dried season as opposed to the works of Omalu et al., [22], and Ejima et al., [23], that were carried out during raining season. This finding agrees with Ayanda [27], who opined that prevalence of* P. falciparum *infection is higher in the wet season than in the dry season, while Minakaw et al., [28], reported that rainy season presents favorable environmental conditions that enhance mosquito breeding and survival, through the proliferation of larval habitats and improved humidity, respectively.

Various researchers have reported high seroprevalence of malaria at different trimesters of pregnancy [29]. However, according to gestational age of pregnancy, this study recorded the highest seroprevalence rate in the third trimester followed by the first trimester and least was recorded among subjects in their second trimester. This finding agrees with the works of Idowu et al., [29], Ejima et al., [23], and Omalu et al., [22], which recorded high seroprevalence in third trimester and least in second trimester, but does not correlate with the work of Brabin, [30], who reported higher prevalence in the second trimester of pregnancy, while Allesandro and Langerock, [31], and Obianumba, [32], identified higher risk of malaria in the first trimester of pregnancy. However, the 3rd trimester being of highest seroprevalence rate of malaria infection as obtained in this study suggests that the pregnant women have significant loss of immunity during late pregnancy.

The notion of pregnancy as an altered state of immune suppression is well documented [33, 34]. Third trimester of pregnancy is a time period that poses a risk of increased susceptibility to parasitic and infectious diseases, since the maternal immune system is solely responsible for defending against infections and protecting the fetus because both the fetal and the placental responses are limited [35]. This may have been the reason for the higher susceptibility to malaria by women in their third trimester of pregnancy, as recorded in this study

Therefore, the pregnant women in this area may have evidence of malarial infection at the time of birth if proper control and preventive measures are not taken, thus increasing the susceptibility of their offspring to incidence of congenital malaria, low birth weight, and mortality. In support of this claim, Omalu et al., [36], reported 2.63% congenital malaria, 4.61% placental malaria, and 5.92% cord malaria with low birth weight but no mortality in this region (Minna, Niger State). Similarly, 5.10% prevalence of congenital malaria was recorded at Ibadan University College Hospital [37]., 46.70% at Ile-Ife, Southwestern Nigeria [38], and 13.00% at Calabar Teaching Hospital [39]. Also, the observation with second trimester could be a result of constant intermittent preventive treatments in pregnancy (IPTp) given to pregnant women during antenatal care visit which usually commence during second trimester [32]

Rapid diagnostic test (RDT) has been recommended to improve diagnostic efficiency, which is important for preventing indiscriminate use of ACT, thereby preventing or delaying the development of parasite resistance to this new first-line drug [40]. Also, RDTs can be used as a stop-gap when microscopy services are not operating or as a primary diagnostic tool for rural/remote areas without microscopy services [41]. The sensitivity of RDT reported in this study (75%) is lower than 100% sensitivity previously reported in Nigeria [42] and 96%, 97%, and 97.6% reported in Zambia, Zanzibar, and Thailand, respectively [40, 43, 44]. However, percentage of patients that were false positive (25%) recorded in this study is considerably high. This suggests that there were chances of RDT to classify a healthy patient as being sick. Ayogu, [45], reported a very low (3.2%) failure of PCR confirmation of malarial patient who tested positive by RDT. The patients with false positive result by RDT are likely to be patients with persistently circulating antigen due to prior use of antimalarial. However, other factors not investigated in this work, such as age differences in sensitivity to RDT, rheumatoid factor cross-reacting in the blood [46], and cross-reactivity with heterophile antibodies [47], can be responsible for false positive result in RDT. It is therefore necessary to consider clinical situations and laboratory and especially microscopic confirmation tests in cases of suspected false positive results for malaria RDT [48].

Prevalence of malaria parasites seemed to be relatively high across a blood group O (53.84%) and AB (7.69%) subjects recording the least infection rates. High prevalence of infection in blood group O shows that they are most susceptible to uncomplicated malaria infection. This disagrees with the findings of Simon-Oke et al., [16], who reported that prevalence of malaria parasites was highest among those with AB blood group (60.0%) and lowest in those with B blood group (37.2%).

Natural selection for resistance against malaria may favor blood group O as it protects against severe* P. falciparum* malaria [49]. However, it should be clear that the high occurrence of malaria in blood group O in this study population is not surprising, as the finding does not disapprove the hypothesis about a selective survival evolutionary advantage of* P. falciparum* infection in blood group O compared with non-O blood groups in malaria endemic areas, but rather further explains the hypothesis that O blood type groups are more susceptible to uncomplicated malaria but less susceptible to complicated malaria. This corresponds to the result of Rowe et al. [8] in Mali, where blood group O had a higher prevalence of uncomplicated malaria.

There are numerous publications on the strong protection of the HbAS trait against mild and severe* falciparum* malaria. However, this study shows that pregnant women with genotype “AA” were most susceptible to malaria with 64.10% of the total infection compared to AS (35.89%). These are not surprising, as similar occurrences have been reported in other parts of Nigeria and other African countries [50–56]. It has been recorded by Friedman, [57], that the lowering of oxygen which causes sickling shape in the blood of SS and AS people reduces the parasite growth and can cause the parasites to die, but this is not so with the AA genotype. Genotype AA is the most prevalent genotype in this part of the world and more prone to malaria infection than other genotypes because of the absence of any sickle cell molecules in the blood.

Anaemia in* falciparum* malaria is mainly due to the destruction of parasitized red cells. However, according to Ogbodo et al., [58], increased parasite density ultimately leads to increase in red cell breakdown and consequently anaemia. Brabin, [30] reported 70–80% of pregnant women in malaria area are susceptible to anaemia. However, based on the definition of anaemia in pregnancy, which is PCV of less than 30% [59], infected women in this study cannot be classified as being anaemic (as the mean PCV was >30%). These findings corroborate with those of similar studies which reported PCV level of 30.16% ± 5.55% [60] and 33.56 ± 0.48% [61] in pregnant women. However, the mean PCV, RBC, and HGB among malaria infected mothers were lower than mean PCV, RBC, and HGB of those without malaria parasite, which can be suggestive of vulnerability to anaemia in pregnancy, thus placing them at higher risk of morbidities such as congestive heart failure, fetal demise, and mortality associated with hemorrhage at the time of delivery [62].

The use of advanced molecular techniques is extremely useful for the detection of drug resistance in malarial parasites and plays an immense role in the epidemiological survey as well as in regular updating of the antimalarial drug policy regimes [4]. Therefore, it plays an important role in hospital-based prevalence study to monitor the drug resistance. Its use in detection of the point mutation in the* pfmdr1* gene in this study reports no mutation detection at codon positions 86, 184, 1034, and 1042 of* pfmdr1* in* P. falciparum* isolates from pregnant women in Minna, Niger State, Northern Nigeria, suggesting that these alleles are not a marker for multidrug resistant* Plasmodium falciparum* in Niger State, Nigeria, but larger sample size should conducted.

*Plasmodium falciparum *multidrug resistance gene 1* (pfmdr1)* is an adenosine triphosphate-binding cassette protein located on the parasite's food vacuole and thus facilitates transport of solutes into the cell. In sensitivity to antimalarial drugs* pfmdr1 *acts as an active drug transporter, thus quinine and possibly other antimalarial drugs occupy the common drug binding site of* pfmdr1*, thereby inhibiting transport of other solutes. Mutations in this gene are known to alter the binding of this drug, thereby allowing the flow of solute via parasite's food vacuole, and thus drug resistance developed [63]. Therefore, the prediction of the structure* pfmdr1* gene is important for identification of an analogous compound which can appropriately fit into the parasite food vacuole and block the flow/movement of solute into the parasite, thereby suggesting new ligand for drug target modification.

Basic local alignment search tool (BLAST) is the tool most frequently used for calculating sequence similarity [64]. The table of BLAST hits is a section showing all of the alignment blocks for each BLAST hit. The sequence alignments show us how well the* pfmdr1* gene isolated matches* pfmdr1* gene reported in the database for other African countries. It has been reported that BLAST results with sequence identity ranges of 75–92% have high degree of sequence homology [65]. In this study, it was noted that the five hits match much better the query* pfmdr1* sequence than the remaining BLAST hits, and the MSA of these genes shows very high similarities of the gene with those reported from other African countries, therefore suggesting that a particular ligand could be predicted to target* pfmdr1* gene in* P. falciparum* there by opening a novel window for malaria drug discovery.

It has been reported that molecular interaction and function of a protein depend on its three-dimensional structure [64]. Thus, prediction of protein structures assists in predicting the chemical ligands/inhibitor that can bind and change the expression or inhibit its expression, thus providing a new window for drug target.

On the basis of this,* pfmdr1* gene was searched for similar protein sequence (homologous protein sequence) and structure prediction through PDBSum databases. However, 4RHS, SPLTB, STT, and 5LUQ are the most similar proteins with 31.7 and 34.8 percentages. T-Coffee and ESPript 3.0 show that there is a good alignment and orientation of* pfmdr1* gene sequence with crystal structure of pltb (SPLTB) and structure of typhoid toxin (STT). This suggested that this gene shares structural identity.

T-Coffee has been reported to be definitely superior to other MSA programs with regard to alignment accuracy in all datasets [66]. Its alignment similarities ranges of 58%–91% have been considered to be of high degree [67]. In this study, alignment of* pfmdr1* shows a high degree of homology to the STT (65%) and SPLTB (64%). IEMDT, however, shows least similarities of 37% (Figure 2). In the 3-dimensional helical folding of the homologous proteins,* IEMDT* shows twenty-one *α*-helices, twelve *β* strands, and two n, while SPLTB and STT have two *α*-helices and five *β* strands (Figure 3). This further removes IEMDT as a strong structural prediction for in* pfmdr1*.

However, it has been reported that typhoid toxin (STT) exhibits strong selectivity for Neu5Ac-terminated glycans, which is predominantly expressed in human cells, over Neu5Gc-terminated glycans, predominantly expressed by most other mammals. This toxin bound a diverse group of sialylated glycans with preferential binding to termini with the consensus sequence Neu5Ac*α*2-3Gal*β*1-3/*β*1-4Glc/GlcNAc. Therefore the exquisite binding selectivity of typhoid toxin for glycans predominantly expressed in human cells provides an explanation for the inability of* S. typhi* to cause typhoid fever in some nonpermissive species like chimpanzees. This is consistent with previous results on crystal structure of pltb (SPLTB) and crystal structure of gd2 bound pltb which show similar structural conformation with* pfmdr1* gene isolated in this study [68]. Since, crystal structure of pltb and typhoid toxin share similar structural conformation with the isolated* pfmdr1* gene in this study, this could also be the reason why* P. falciparum* causes malaria only in humans. Conclusively, this compound (Neu5Ac*α*2-3Gal*β*1-3/*β*1-4Glc/GlcNAc) can theoretically bind and change the functional integrity of the pfmdr1 protein, thus providing a new window for drug target.

## Figures and Tables

**Table 1:** Prevalence of malaria infection among different gestation periods of pregnant women attending General Hospital Minna.

Stages of pregnancy	Frequency (%)
1st trimester	12 (30.76)
2nd trimester	9 (23.07)
3rd trimester	18 (46.15)
*t*-value	4.91
*p*-value	0.039

**Table 2:** Prevalence of malaria infection among different blood groups of pregnant women attending General Hospital Minna.

ABO blood groups	Frequency (%)
A	5 (17.24)
B	10 (25.64)
AB	3 (7.69)
O	21 (53.84)
*t*-value	2.42
*p*-value	0.094

**Table 3:** Prevalence of malaria infection among different genotype of pregnant women attending General Hospital Minna.

Genotype	Frequency (%)
AA	25 (64.10)
AS	14 (35.89)
SS	0 (0.0)
AC	0 (0.0)
*t*-value	1.609
*p*-value	0.206

**Table 4:** Hematological parameters in *Plasmodium falciparum* infected pregnant women attending General Hospital Minna.

Subjects	PCV (%)	HGB (g/l)	RBC	WBC (×10^9^/L)	Platelet (×10^3^/L)
Malaria Positive (*N* = 20)	33.56 ± 4.01	8.93 ± 1.09	5.97 ± 0.68	4.80 ± 0.32	267.89 ± 26.90
Malaria Negative (*N* = 10)	44.50 ± 3.20	9.03 ± 1.47	7.01 ± 1.76	4.20 ± 0.45	598.67 ± 39.77
*p*-value	0.0243	0.195	0.194	0.271	0.007

Red blood cells (RBC), haemoglobin (HGB), packed cell volume (PCV), total white blood cell (WBC), and differential counts.

**Table 5:** Protein sequence of* Pfmdr1* gene isolates from genomic DNA of *P. falciparum* infected pregnant patient sample.

Sample code	Primers used	Protein sequence	S1034C	N1042D
A1	F: AGGTTGAAAAAGAG AAC and R: ATGACACCACAAACATAA	GTDYFCNLIEKAIDYKNKGQKRRIIVNAALWGFSQSAQLFINSFAYWFGSXLIKRGTILVDDFMKXX	W	W

A3	F: AGGTTGAAAAAGAG AAC and R: ATGACACCACAAACATAA	DYFCI*∗∗*KKLLIIKIKDKKEELL*∗*MQLYGDSVKALNYLLIVLPIGLDPS*∗*LKEVLY*∗*LMT L*∗*NPDVFT*∗*X	D	D

W: wild type; D: disrupted protein sequence.

**Table 6:** Amino acid and translated protein sequence of the *pfmdr1* gene fragments.

Amino acid sequence	Translated protein sequence	Blastp result
GGCACAGATTATTTCTGTAATTTGATAGAAAAAGCTATTGATTATAAAAATAAAGGACAAAAAAGAAGAATTATTGTAAATGCAGCTTTATGGGGATTCAGTCAAAGCGCTCAATTATTTATTAATAGTTTTGCCTATTGGTTTGGATCCWTCTTAATTAAAAGAGGTACTATATTAGTTGATGACTTTATGAAATSCA	GTDYFCNLIEKAIDYKNKGQKRRIIVNAALWGFSQSAQLFINSFAYWFGSXLIKRGTILVDDFMKXX	BLASTP result was used for multiple sequence alignment and further bioinformatics data search

GATTATTTCTGTATTTGATAGAAAAAGCTATTGATTATAAAAATAAAGGACAAAAAAGAAGAATTATTGTAAATGCAGCTTTATGGGGATTCAGTCAAAGCGCTCAATTATTTATTAATAGTTTTGCCTATTGGTTTGGATCCTTCTTAATTAAAAGAGGTACTATATTAGTTGATGACTTTATGAAATCCAGATGTTTTTACATGAGA	DYFCI*∗∗*KKLLIIKIKDKKEELL*∗*MQLYGDSVKALNYLLIVLPIGLDPS*∗*LKEVLY*∗*LMT L*∗*NPDVFT*∗*X	No data found

**Table 7:** PDBSum database information of *pfmdr1* gene homologous protein sequence. *Sequence*: GTDYFCNLIEKAIDYKNKGQKRRIIVNAALWGFSQSAQLFINSFAYWFGSXLIKRGTILVDDFMKXX. Sequence length: **67** residues.

	PDB code	Model	Length	%-tage identity	a.a. overlap	*z*-score	Ligands	Protein name
1	3wmg(A)	X-ray 2.40 Å	589	27.3%	44	139.8	DMU, TRS.	Crystal structure of an inward-facing eukaryotic ABC multidrug transporter g277v/a278v/a279v mutant in complex with a cyclic peptide inhibitor, aCAP

2	3wmf(A)	X-ray 2.60 Å	588	27.3%	44	139.8	DMU.	Crystal structure of an inward-facing eukaryotic ABC multidrug transporter g277v/a278v/a279v mutant

3	IEMDT(A)	X-ray 2.75 Å	588	27.3%	44	139.8	DMU.	Crystal structure of an inward-facing eukaryotic ABC multidrug transporter

4	4rhs(A)	X-ray 1.92 Å	114	31.7%	41	126.9	ACT, SIA-SIA-GAL.	Crystal structure of GD2 bound pltb

5	SPLTB(A)	X-ray 2.08 Å	114	31.7%	41	126.9	ACT.	Crystal structure of pltb

6	STT(A)	X-ray 2.39 Å	114	31.7%	41	126.9	GOL.	Structure of typhoid toxin

7	5luq(A)	X-ray 4.30 Å	3725	34.8%	46	111.6		Crystal structure of human DNA-dependent protein kinase catalytic subunit (DNA-PKcs)
